# Knowledge, attitudes, and practices of lung cancer patients regarding nutritional management during chemotherapy

**DOI:** 10.3389/fnut.2026.1678612

**Published:** 2026-01-21

**Authors:** Jie Cao, Feng Xiang, Jun Zhang, Yun Tang, Min Hu, Fangfei Li

**Affiliations:** 1 Department of Traditional Chinese Medicine Oncology, Chongqing University Cancer Hospital, Chongqing, China; 2 Chongqing Key Laboratory for the Mechanism and Intervention of Cancer Metastasis, Chongqing, China

**Keywords:** chemotherapy, cross-sectional studies, knowledge, attitudes, practice, lung cancer, nutrition therapy

## Abstract

**Objective:**

Malnutrition is common in lung cancer patients receiving chemotherapy and contributes to treatment intolerance, poorer quality of life, and reduced survival, emphasizing the need for effective nutritional management. This study aims to investigate the knowledge, attitudes, and practices (KAP) related to nutritional management during chemotherapy among lung cancer patients.

**Methods:**

A cross-sectional survey was conducted at Chongqing University Cancer Hospital between May and July 2025. Patients with lung cancer were invited to complete a structured questionnaire to collect demographic data and evaluate KAP related to nutritional management during chemotherapy. The Nutritional Risk Screening 2002 score (NRS-2002) was used to identify individuals at risk of malnutrition.

**Results:**

A total of 598 valid responses were collected. Most participants were male (57.4%). More than half of the patients (57.4%) had a history of chemotherapy, and 14.1% had an NRS-2002 score above 3, indicating nutritional risk. The mean scores for knowledge, attitude, and practice were 12.07 ± 4.69 (range: 0–20), 35.89 ± 4.98 (range: 9–45), and 23.28 ± 4.12 (range: 6–30), respectively. Structural equation modeling revealed that knowledge had significant direct effects on both attitude (*β* = 0.601, *p* = 0.011) and practice (*β* = 0.345, *p* = 0.006). Attitude also directly influenced practice (*β* = 0.558, *p* = 0.010). Additionally, knowledge exerted an indirect effect on practice through attitude (*β* = 0.335, *p* = 0.014).

**Conclusion:**

Patients with lung cancer undergoing chemotherapy demonstrated limited knowledge but generally positive attitudes and moderate practices regarding nutritional management. Targeted educational interventions are warranted to enhance patients’ nutritional knowledge, which may further reinforce their attitudes and improve their self-management behaviors during chemotherapy.

## Introduction

Lung cancer remains a formidable global health challenge, representing the leading cause of cancer-related mortality worldwide. According to the latest GLOBOCAN 2022 estimates, approximately 2,480,675 new cases and 1,817,469 deaths from lung cancer occurred globally in 2022, accounting for 12.4% of all cancer diagnoses and 18.7% of all cancer deaths (1). In China alone, over 1.06 million new lung cancer cases were diagnosed, representing more than 40% of the global burden (2). Chemotherapy remains one of the primary treatment modalities for lung cancer. Approximately 55–70% of patients with advanced non-small cell lung cancer (NSCLC) receive chemotherapy or systemic therapy as part of their initial treatment in current clinical practice (3, 4). However, treatment-related side effects such as nausea, vomiting, loss of appetite, and altered taste perception frequently compromise patients’ nutritional intake and overall treatment tolerance (5).

Malnutrition represents a critical concern among lung cancer patients, with prevalence rates ranging from 35 to 70% depending on treatment modality, disease stage, and assessment methods (6). This condition is associated with adverse outcomes, including reduced survival, poorer quality of life, and impaired physical function (7). Li et al. (8) have reported that malnutrition, defined by the Global Leadership Initiative on Malnutrition (GLIM) criteria, significantly increased chemotherapy-related toxicities in patients with advanced NSCLC. Almuradova and Menekse (9) have demonstrated that a lower prognostic nutritional index was independently associated with shorter overall survival in elderly patients with small-cell lung cancer. Similarly, Taranova et al. (10) have found that poorer patient-reported nutritional status was associated with greater treatment toxicity and worse survival in patients with limited-stage small cell lung cancer. Moreover, chemotherapy may exacerbate the severity of malnutrition by inducing gastrointestinal toxicity, reducing appetite, and increasing metabolic demands (11). The knowledge, attitude, and practice model provides a theoretical framework for understanding health-related behaviors, postulating that individual practices are contingent upon one’s knowledge and attitudes (12). This theoretical framework is particularly valuable for assessing and improving health-related behaviors in cancer patients, as studies have demonstrated that patient education can significantly impact treatment outcomes, including nutritional intake and psychological status (13).

While evidence demonstrates the effectiveness of nutritional education and counseling interventions in improving outcomes for cancer patients (13), research specifically examining KAP regarding nutritional management among lung cancer patients remains limited. Recent studies have further highlighted the issue of nutrition management in cancer patients. For example, Zhi et al. (14) explored the KAP regarding nutritional management among patients with gastrointestinal cancer and found considerable gaps, further supporting the necessity and innovation of focusing on lung cancer patients during chemotherapy. A recent cross-sectional study investigating knowledge, attitudes, and behaviors toward healthy eating among Chinese cancer patients treated with chemotherapy found that patients often had contradictory and confused information about nutrition, with knowledge, attitudes, and behaviors toward healthy eating being “not satisfactory and need to be improved” (15). Given that inadequate nutritional knowledge and attitudes may lead to poor dietary behaviors, which can exacerbate malnutrition and adversely affect treatment tolerance, quality of life, and prognosis, understanding patients’ nutritional KAP is a crucial first step. However, no studies have specifically investigated the comprehensive nutritional KAP status among Chinese lung cancer patients.

Therefore, this study aims to investigate the current status of knowledge, attitudes, and practices regarding nutritional management during chemotherapy among lung cancer patients in China.

## Materials and methods

### Study design and participants

A cross-sectional survey was carried out between May and July 2025 at Chongqing University Cancer Hospital, targeting individuals diagnosed with lung cancer. The study protocol received approval from the Medical Ethics Committee of Chongqing University Cancer Hospital (approval no.: CZLS2025143-A), and informed consent was obtained from all participants prior to data collection.

Participants were eligible for inclusion if they met the following criteria: (1) Histopathologically confirmed diagnosis of lung cancer; (2) had received or were scheduled to receive chemotherapy; (3) aged 18 years or older; and (4) fully informed about the study’s objectives and willing to participate voluntarily. Exclusion criteria included the presence of comorbid conditions that could significantly interfere with nutritional status, such as gastrointestinal obstruction or advanced hepatic or renal insufficiency. During routine clinical care, attending physicians provided standard nutrition education, including guidance on maintaining adequate caloric and protein intake, achieving dietary balance, managing chemotherapy-related side effects, and regular weight monitoring. To ensure that our sample reflects the general population of lung cancer patients, participants in this study did not receive any additional or specialized nutrition education beyond routine clinical guidance.

### Questionnaire

The questionnaire was developed with reference to existing literature (14, 15) and relevant clinical guidelines, including the Nutrition Guide for Lung Cancer Patients (16), the Guidelines for nutritional management in patients undergoing chemotherapy (17), and the Expert consensus on nutritional treatment for patients with gastroparesis (18). The initial version underwent expert review and was subsequently revised to enhance clarity and alignment with study objectives. Specifically, the questionnaire was reviewed by an oncology expert with over 20 years of clinical experience and a nutrition expert with 17 years of professional background. Based on their recommendations, the study was revised to include tumor staging information and additional nutritional indicators. They also advised streamlining the questionnaire to improve its feasibility and respondent compliance. A pilot test was conducted with 36 participants to assess the instrument’s psychometric properties. Reliability analysis yielded a high overall internal consistency, with a Cronbach’s alpha of 0.895. Subscale coefficients were also acceptable: 0.887 for the knowledge section, 0.788 for the attitude section, and 0.809 for the practice section.

The finalized questionnaire, administered in Chinese, comprised four sections: (1) demographic information—including age, sex, education level, residential location, marital status, monthly income, time since diagnosis, treatment modality, height, weight, NRS-2002, and family history of lung cancer; (2) knowledge dimension; (3) attitude dimension; and (4) practice dimension. Body mass index (BMI) was calculated as BMI = weight (kg)/height (m^2^). NRS-2002 is a validated tool used to identify individuals at risk of malnutrition (19). It assesses both nutritional status and disease severity, which are key determinants of a patient’s nutritional needs. The screening comprises three components: an initial set of screening questions, an evaluation of nutritional status (including weight loss, body mass index, and dietary intake), and an assessment of disease severity. The total score ranges from 0 to 7, with a score ≥3 indicating a risk of malnutrition. The knowledge section consisted of 10 items, with response options scored as follows: “very familiar” = 2 points, “heard of it” = 1 point, and “not clear” = 0 points, producing a possible total score ranging from 0 to 20. The attitude section comprised 9 items evaluated using a 5-point Likert scale, with responses ranging from “strongly agree” (5 points) to “strongly disagree” (1 point), yielding a total score range of 9 to 45. The practice section included 6 items, each scored from 1 to 5, reflecting frequency from “rarely” to “always,” with total scores ranging from 6 to 30. Two additional multiple-choice items related to practice (items 7 and 8) were not scored using this scale and were instead analyzed descriptively. For interpretive purposes, Bloom’s cut-off value, which has been widely used in public health and nutrition-related KAP studies (20–22), was adopted to categorize scores as good, moderate, or poor: scores of 80% or above of the maximum possible score were considered good; scores between 60 and 79% were considered moderate; and scores below 60% were classified as poor (23, 24).

### Questionnaire distribution and quality control

A convenience sampling method was employed in this study, with data collected through an online electronic questionnaire. The questionnaire was developed using the Wenjuanxing platform^1^ and a corresponding QR code was generated for distribution. This QR code was shared with lung cancer patients in the oncology and thoracic surgery departments via WeChat for data collection. The study objectives, privacy protection measures, and the electronic survey link were also disseminated through WeChat support groups for lung cancer patients to invite participation. During the questionnaire completion process, research assistants provided necessary explanations to ensure participants fully understood the content and purpose of the study. Upon completion of data collection, members of the research team conducted quality control assessments. Questionnaires were deemed invalid and excluded if they met any of the following criteria: completion time less than 60 s, entry of abnormal numerical values, incorrect responses to attention-check (trap) questions, or detection of logical inconsistencies or repetitive response patterns.

### Sample size

The sample size for this study was calculated based on a previously published method (14) using the formula: *n* = (*z*^2^·*p*·*q*)/*e*^2^, where *n* is the required sample size, *z* represents the standard normal deviate for a 95% confidence level (1.96), *p* is the expected proportion, *q* = 1 − *p*, and *e* is the margin of error set at 5%. A conservative estimate of *p* = 0.5 was used to yield the maximum sample size. Based on this calculation, the minimum required sample size was 384. To account for potential invalid responses, an additional 20% was added, resulting in a final target sample size of 480 participants.

### Statistical analysis

Statistical analyses were performed using SPSS version 26.0 (IBM Corp., Armonk, NY, United States) and AMOS version 24.0 (IBM Corp., Armonk, NY, United States). The continuous variables were described as means ± standard deviations (SD). The categorical variables were described as *n* (%). The normal distribution of continuous data was checked using the Kolmogorov–Smirnov test. The continuous variables conforming to the normal distribution analyzed using Student’s *t*-test or ANOVA. Those with a skewed distribution were analyzed using the Wilcoxon-Mann–Whitney *U*-test or the Kruskal–Wallis analysis of variance. Construct validity of the questionnaire was evaluated using Confirmatory Factor Analysis (CFA), and sampling adequacy was confirmed with the Kaiser-Meyer-Olkin (KMO) test. Model fit for both the CFA and subsequent structural equation modeling (SEM) was evaluated using the Root Mean Square Error of Approximation (RMSEA), Standardized Root Mean Square Residual (SRMR), Tucker–Lewis Index (TLI), and Comparative Fit Index (CFI). Acceptable model fit was defined as RMSEA and SRMR values <0.08, and TLI and CFI values >0.80. Spearman’s correlation was used to determine the correlation among the KAP score. To identify predictors of KAP scores, univariate and multivariate linear regression analyses were performed. Variables with *p* < 0.05 in univariate analysis were entered into the multivariate model to determine independent associations. SEM was conducted to assess the interrelationships among the KAP components, based on the theoretical framework of KAP study. In particular, the mediating role of attitude in the pathway from knowledge to practice was tested. Both direct and indirect effects were estimated and compared. All statistical tests were two-sided, and a *p*-value of less than 0.05 was considered indicative of statistical significance.

## Results

Initially, a total of 734 questionnaires were collected. The following responses were excluded: 6 cases declined participation, 3 cases had response times less than 60 s, 3 cases were from individuals under 18 years old, 3 cases had abnormal entries, 3 cases had outlier height values, and 118 cases failed the trap question. After exclusions, 598 valid questionnaires were retained, with an effective rate of 81.47%.

### Demographic information on participants

Regarding clinical characteristics, 21.1% of patients had been diagnosed within 6 months, while 34.9% had been diagnosed for more than 1 year. Approximately half of the patients (51.7%) had undergone surgery, and 48.0% had received radiotherapy. More than half of the patients (57.4%) had a history of chemotherapy before the current cycle. In terms of nutritional status, 14.1% had an NRS-2002 score above 3, indicating nutritional risk. Additionally, 30.1% reported a family history of lung cancer among first-degree relatives. Their mean knowledge, attitude, and practice scores were 12.07 ± 4.69 (possible range: 0–20), 35.89 ± 4.98 (possible range: 9–45), and 23.28 ± 4.12 (possible range: 6–30), respectively. Knowledge scores were significantly higher among urban residents, married individuals, those with longer disease duration, prior chemotherapy, and those who received nutritional education. Attitude scores were significantly associated with residence, education level, surgery history, radiotherapy status, BMI, and nutritional education. Practice scores showed significant associations with residence, marital status, disease duration, radiotherapy status, BMI, family history, nutritional education, and nutritional risk (all *p* < 0.05) (Table 1). CFA supported the structural validity of the KAP questionnaire, and sampling adequacy was confirmed by the KMO test (KMO = 0.937, *p* < 0.001) (Supplementary Figure S1 and Supplementary Tables S1,S2).

**Table 1:** Demographic characteristics and KAP scores.

Variables	*N* (%)	Knowledge (mean ± SD)	*p* for knowledge	Attitude (mean ± SD)	*p* for attitude	Practice (mean ± SD)	*p* for practice
*N* = 598		12.07 ± 4.69		35.89 ± 4.98		23.28 ± 4.12	
Age (years)							
18–39	160 (26.76)	11.86 ± 4.11	0.620	35.32 ± 6.04	0.982	22.59 ± 4.17	0.065
40–60	228 (38.13)	12.17 ± 4.55		36.14 ± 4.36		23.45 ± 3.75	
61 and more	210 (35.12)	12.12 ± 5.24		36.05 ± 4.71		23.63 ± 4.39	
Gender							
Male	343 (57.36)	12.03 ± 4.85	0.804	35.97 ± 4.7	0.617	23.23 ± 4.08	0.364
Female	255 (42.64)	12.12 ± 4.48		35.78 ± 5.34		23.35 ± 4.17	
Residence							
Rural	228 (38.13)	11.32 ± 4.62	0.002	35.44 ± 4.24	0.003	22.62 ± 3.95	0.001
Urban	370 (61.87)	12.53 ± 4.69		36.16 ± 5.38		23.69 ± 4.16	
Education							
Junior high school and below	221 (36.96)	11.63 ± 5.05	0.453	35.92 ± 4.57	0.008	23.1 ± 4.36	0.887
High school/technical secondary school	134 (22.41)	12.31 ± 4.67		35.66 ± 4.31		23.53 ± 3.84	
Associate degree	110 (18.39)	11.96 ± 4.88		34.74 ± 6.66		23 ± 4.77	
Bachelor’s degree and above	133 (22.24)	12.65 ± 3.83		37.02 ± 4.43		23.57 ± 3.32	
Marital status							
Unmarried	127 (21.24)	10.7 ± 4.4	<0.001	34.91 ± 6.16	0.152	21.91 ± 4.58	<0.001
Married	471 (78.76)	12.44 ± 4.7		36.15 ± 4.58		23.65 ± 3.9	
Monthly income per capita (CNY)							
<2,000	84 (14.05)	11.4 ± 5.47	0.254	35.4 ± 4.37	0.171	23.12 ± 4.22	0.131
2,000–5,000	285 (47.66)	12.24 ± 4.47		36.07 ± 4.42		23.56 ± 3.96	
5,000–10,000	185 (30.94)	11.86 ± 4.66		35.66 ± 5.61		22.77 ± 4.22	
>10,000	44 (7.36)	13.07 ± 4.56		36.59 ± 6.53		23.93 ± 4.31	
Time since lung cancer diagnosis							
Within 1 month	73 (12.21)	11.01 ± 4.34	0.004	35.64 ± 4.7	0.596	22.07 ± 4.18	<0.001
1–3 months	68 (11.37)	11.69 ± 4.35		35.97 ± 4.39		22.85 ± 3.78	
3–6 months	122 (20.4)	11.86 ± 4.45		35.37 ± 5.55		22.46 ± 3.94	
6 months−1 year	126 (21.07)	11.74 ± 4.69		35.53 ± 5.57		22.95 ± 4.44	
1–3 years	131 (21.91)	12.47 ± 4.88		36.23 ± 4.2		24.42 ± 3.63	
More than 3 years	78 (13.04)	13.56 ± 5.06		36.86 ± 4.95		24.71 ± 4.09	
History of surgery							
Yes	309 (51.67)	12.31 ± 4.43	0.152	36.42 ± 4.55	0.008	23.37 ± 3.93	0.775
No	289 (48.33)	11.81 ± 4.96		35.32 ± 5.35		23.19 ± 4.3	
History of radiotherapy							
Yes	287 (47.99)	11.91 ± 4.38	0.375	35.38 ± 5.42	0.035	22.74 ± 4.24	0.003
No	311 (52.01)	12.22 ± 4.96		36.36 ± 4.5		23.78 ± 3.94	
History of chemotherapy							
Yes	343 (57.36)	12.68 ± 4.62	<0.001	36.16 ± 4.74	0.287	23.5 ± 4.09	0.116
No	255 (42.64)	11.25 ± 4.68		35.52 ± 5.28		22.99 ± 4.14	
BMI							
<18.5	100 (16.72)	12.42 ± 4.34	0.139	37 ± 4.52	0.003	24.19 ± 3.69	0.014
18.5–24.9	355 (59.36)	12.22 ± 4.66		35.9 ± 5.07		23.22 ± 4.12	
>24.9	143 (23.91)	11.45 ± 4.98		35.08 ± 4.95		22.8 ± 4.31	
Family history of lung cancer (first-degree relatives)							
Yes	180 (30.1)	12.32 ± 4.17	0.492	35.62 ± 4.54	0.125	22.85 ± 3.7	0.029
No	418 (69.9)	11.96 ± 4.9		36 ± 5.16		23.47 ± 4.27	
Education from a hospital or other healthcare institutions							
Yes	366 (61.2)	13 ± 3.96	<0.001	36.87 ± 4.19	<0.001	23.77 ± 3.63	0.002
No	232 (38.8)	10.6 ± 5.34		34.33 ± 5.69		22.51 ± 4.69	
NRS-2002							
≤3	514 (85.95)	12.14 ± 4.71	0.397	36.13 ± 4.79	0.062	23.53 ± 4.01	<0.001
>3	84 (14.05)	11.63 ± 4.57		34.4 ± 5.85		21.75 ± 4.4	

Normality testing indicated that continuous variables were skewedly distributed, and appropriate non-parametric tests were applied where required.

### Knowledge, attitude, and practice

The distribution of knowledge dimension revealed that the three questions with the lowest number of participants choosing the “Very familiar” option were “The recommended energy intake target for lung cancer patients is 25–30 kcal per kilogram of body weight per day.” (K4) with 24.25%, “When patients cannot obtain adequate nutrition through enteral nutrition (e.g., severe radiation esophagitis, severe nausea and vomiting), parenteral nutrition should be chosen.” (K10) with 30.1%, and “Chemotherapy patients with good nutritional status and no nutritional risk do not require routine nutritional management.” (K6) with 30.6% (Table 2). These findings indicate that patients were less familiar with technically specific aspects of nutritional management, such as caloric estimation and indications for parenteral nutrition, which may exceed the level of knowledge typically expected from patients undergoing chemotherapy.

**Table 2:** Distribution of knowledge dimension responses.

Knowledge items [*n* (%)]	Very familiar	Heard of it	Not clear	*p* ^1^	*p* ^2^	*p* ^3^
1. Chemotherapy for lung cancer often causes nausea, vomiting, diarrhea, taste changes, loss of appetite, and anorexia, and may even cause liver damage. These effects ultimately impact nutrient intake and easily lead to malnutrition.	257 (42.98)	309 (51.67)	32 (5.35)	0.001	0.014	0.150
2. Malnutrition reduces patients’ tolerance to chemotherapy, affects quality of life, treatment effectiveness, and prognosis, and increases the likelihood of adverse chemotherapy reactions.	257 (42.98)	273 (45.65)	68 (11.37)	0.093	0.967	0.230
3. Lung cancer patients need to pay attention to balanced nutrition and additional nutritional supplementation throughout the entire treatment process.	261 (43.65)	253 (42.31)	84 (14.05)	0.220	<0.001	0.140
4. The recommended energy intake target for lung cancer patients is 25–30 kcal per kilogram of body weight per day.	145 (24.25)	268 (44.82)	185 (30.94)	<0.001	<0.001	0.210
5. Lung cancer patients should increase their protein intake additionally.	232 (38.8)	279 (46.66)	87 (14.55)	<0.001	0.698	0.039
6. Chemotherapy patients with good nutritional status and no nutritional risk do not require routine nutritional management.	183 (30.6)	282 (47.16)	133 (22.24)	0.736	0.024	0.906
7. Nutritional management should be started promptly for patients with moderate to severe malnutrition.	213 (35.62)	288 (48.16)	97 (16.22)	0.191	0.073	0.883
8. For lung cancer patients who are able to eat, autonomous eating should be encouraged as a priority.	256 (42.81)	273 (45.65)	69 (11.54)	0.005	0.362	0.083
9. When a normal diet cannot meet patients’ nutritional needs, additional nutritional supplements can be used.	209 (34.95)	301 (50.33)	88 (14.72)	0.293	0.001	0.549
10. When patients cannot obtain adequate nutrition through enteral nutrition (e.g., severe radiation esophagitis, severe nausea and vomiting), parenteral nutrition should be chosen.	180 (30.1)	305 (51)	113 (18.9)	0.266	<0.001	0.374

*p*^1^, Comparison between different age groups; *p*^2^, Education, *p*^3^, Nutritional risk status (based on NRS-2002 score).

Regarding related attitudes, 18.73% were very worried and 47.66% were worried that they might be malnourished (A1), and 30.94% were very worried and 47.66% were worried that malnutrition might affect their chemotherapy outcomes (A3). On the other hand, 22.07% were neutral on the use of parenteral nutrition (A7) and 21.07% were neutral on their willingness to invest time and money for better nutritional management (A9) (Table 3). These neutral responses may reflect patients’ limited understanding of parenteral nutrition, its perceived invasiveness, and the additional time or financial burden associated with intensive nutritional care during chemotherapy.

**Table 3:** Distribution of attitude dimension responses.

Attitude items [*n* (%)]	Strongly agree	Agree	Neutral	Disagree	Strongly disagree	*p* ^1^	*p* ^2^	*p* ^3^
1. I often worry that I might be malnourished.	112 (18.73)	285 (47.66)	113 (18.9)	76 (12.71)	12 (2.01)	0.058	<0.001	0.220
2. I believe that additional nutritional supplementation is necessary for general lung cancer patients.	207 (34.62)	267 (44.65)	98 (16.39)	21 (3.51)	5 (0.84)	0.221	0.277	0.081
3. I think nutritional management during treatment is very important for chemotherapy success.	202 (33.78)	282 (47.16)	89 (14.88)	20 (3.34)	5 (0.84)	0.014	0.011	<0.001
4. I am concerned that malnutrition will affect my chemotherapy outcomes.	185 (30.94)	285 (47.66)	92 (15.38)	26 (4.35)	10 (1.67)	0.102	0.308	0.083
5. I am willing to undergo regular nutritional assessments.	184 (30.77)	279 (46.66)	111 (18.56)	21 (3.51)	3 (0.5)	0.872	0.045	0.001
6. I am willing to follow dietary restrictions as advised by doctors or nutritionists.	181 (30.27)	278 (46.49)	104 (17.39)	31 (5.18)	4 (0.67)	0.965	0.051	0.272
7. If diet and enteral nutrition alone cannot meet my nutritional needs, I am willing to receive parenteral nutrition.	152 (25.42)	281 (46.99)	132 (22.07)	26 (4.35)	7 (1.17)	0.207	0.874	0.722
8. I believe that good nutritional management can reduce the side effects caused by chemotherapy.	192 (32.11)	299 (50)	83 (13.88)	21 (3.51)	3 (0.5)	0.326	0.059	0.006
9. I am willing to invest time and money to achieve better nutritional management.	150 (25.08)	296 (49.5)	126 (21.07)	25 (4.18)	1 (0.17)	0.929	0.095	0.166

*p*^1^, Comparison between different age groups; *p*^2^, Education; *p*^3^, Nutritional risk status (based on NRS-2002 score).

Responses to the practice dimension showed that 10.54% rarely and 1.67% never used nutritional supplements to help manage the side effects of chemotherapy (P5), and 10.7% rarely and 0.5% never maintained a certain amount of physical activity by doing appropriate activities every day (P6) (Table 4). The low adherence to supplement use and physical activity highlights the need for targeted education and supportive interventions to enhance nutritional self-management during chemotherapy. During treatment, the most commonly reported barriers to nutritional management were limited knowledge (72.6%), complexity and time constraints (64.7%), and appetite-related issues (51.7%), while financial burden was noted by 29.3% of participants. Regarding information sources, the majority relied on physicians (73.9%), followed by new media platforms (66.6%) and hospital-based education (55.5%). Fewer participants reported learning through traditional media (47.8%) or personal networks (51.2%).

**Table 4:** Distribution of practice dimension responses.

Practice items [*n* (%)]	Always	Often	Sometimes	Rarely	Never	*p* ^1^	*p* ^2^	*p* ^3^
1. I adjust my diet according to the recommendations of doctors or nutritionists.	169 (28.26)	248 (41.47)	138 (23.08)	37 (6.19)	6 (1)	0.853	0.355	0.013
2. I regularly measure my weight and pay attention to changes in my body.	163 (27.26)	219 (36.62)	160 (26.76)	47 (7.86)	9 (1.51)	0.454	0.035	0.016
3. In daily life, I pay attention to sufficient nutritional intake and ensure adequate consumption of protein-rich foods.	212 (35.45)	226 (37.79)	125 (20.9)	32 (5.35)	3 (0.5)	0.071	0.860	<0.001
4. I pay attention to a balanced diet and ensure food variety.	219 (36.62)	221 (36.96)	123 (20.57)	34 (5.69)	1 (0.17)	0.009	0.525	<0.001
5. I use nutritional supplements to help manage the side effects of chemotherapy.	161 (26.92)	206 (34.45)	158 (26.42)	63 (10.54)	10 (1.67)	0.177	0.113	0.059
6. I maintain regular physical activity and engage in an appropriate amount of exercise daily.	155 (25.92)	222 (37.12)	154 (25.75)	64 (10.7)	3 (0.5)	0.005	0.051	0.123

*p*^1^, Comparison between different age groups; *p*^2^, Education; *p*^3^, Nutritional risk status (based on NRS-2002 score).

### Subgroup analyses

Because age, education level, and nutritional risk were associated with overall KAP performance, further subgroup analyses were conducted to identify item-level differences (Tables 2–4). Regarding knowledge items, different familiarity was observed in chemotherapy-related adverse effects (K1), energy and protein intake requirements (K4, K5), and the need to prioritize autonomous eating (K8). They also exhibited different recognition of nutritional importance (A3) and varied adherence to balanced diet and exercise behaviors (P4, P6). Participants with lower education levels, particularly those without a bachelor’s degree, tended to have lower scores in understanding treatment-related malnutrition and dietary balance (K1, K3, K4, K9, and K10), as well as less positive attitudes toward nutritional awareness (A1), importance (A3), and regular assessment (A5), and less consistent weight monitoring behaviors (P2), consistent with the overall lower attitude scores seen in Table 1. Patients at nutritional risk (NRS-2002 > 3) also showed lower mean scores for knowledge of protein supplementation (K5), attitudes toward nutritional importance and management (A3, A8) and assessment willingness (A5), and practices related to diet adjustment, weight monitoring, and balanced nutrition (P1–P4). These findings indicate that differences in nutritional knowledge, attitudes, and practices may exist across age, education, and nutritional risk groups.

### Correlations between KAP

In the correlation analysis, significant positive correlations were found between knowledge and attitude (*r* = 0.520, *p* < 0.001), knowledge and practice (*r* = 0.570, *p* < 0.001), as well as attitude and practice (*r* = 0.546, *p* < 0.001), respectively (Supplementary Table S3).

### Interactions between KAP

**Figure 1:**
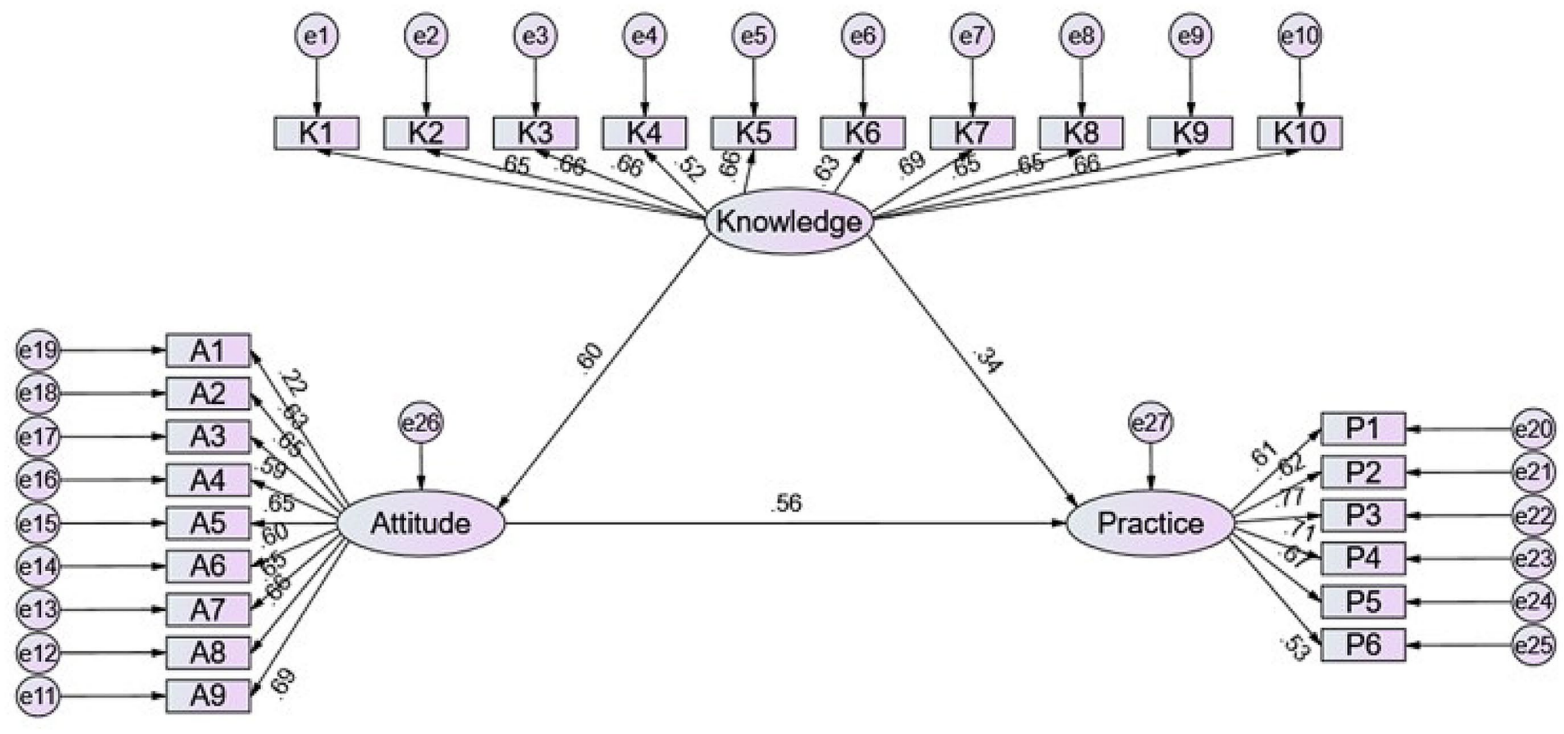
SEM analysis.

The SEM demonstrated a highly favorable model fit indices (CMIN/DF value: 2.823, RMSEA value: 0.055, IFI value: 0.910, TLI value: 0.900, and CFI value: 0.910), suggesting a well-fitting model (Supplementary Table S4). The results of the SEM analysis showed that knowledge had direct effects on attitude (*β* = 0.601, *p* = 0.011) and practice (*β* = 0.345, *p* = 0.006). Meanwhile, attitude had a direct impact on practice (*β* = 0.558, *p* = 0.010). Furthermore, knowledge indirectly affected practice through attitude (*β* = 0.335, *p* = 0.014) (Table 5 and Figure 1).

**Table 5:** SEM direct and indirect effects.

Model paths	Standardized total effects		Standardized direct effects		Standardized indirect effects	
	*β* (95% CI)	*p*	*β* (95% CI)	*p*	*β* (95% CI)	*p*
Knowledge → Attitude	0.601 (0.528–0.671)	0.011	0.601 (0.528–0.671)	0.011		
Knowledge → Practice	0.680 (0.598–0.748)	0.007	0.345 (0.233–0.482)	0.006		
Attitude → Practice	0.558 (0.428–0.648)	0.010	0.558 (0.428–0.648)	0.010		
Knowledge → Practice					0.335 (0.242–0.409)	0.014

### Factors associated with nutritional knowledge, attitude, and practice

To identify determinants of patients’ nutritional KAP scores, univariate and multivariate linear regression analyses were performed. In the multivariate model, higher knowledge scores were associated with higher education, being married, longer disease duration, prior chemotherapy, and receiving nutrition education from healthcare institutions (all *p* < 0.05; Supplementary Table S5). Positive attitude scores were linked to higher knowledge, absence of radiotherapy, prior nutritional education, and lower nutritional risk (all *p* < 0.05; Supplementary Table S6). Better practice scores were related to higher knowledge and attitude levels, urban residence, longer disease duration, and absence of radiotherapy (all *p* < 0.05; Supplementary Table S7). Collectively, these findings indicate that educational attainment, treatment experience, and structured nutritional guidance substantially influence patients’ nutritional KAP, emphasizing the importance of targeted education for individuals with limited schooling, early disease stage, or insufficient professional counseling.

## Discussion

Patients with lung cancer undergoing chemotherapy exhibited insufficient knowledge but generally positive attitudes and moderate practices toward nutritional management. Targeted nutritional education strategies are warranted to enhance patients’ knowledge, which may further reinforce attitudes and improve self-management practices during chemotherapy.

Our findings revealed that patients with lung cancer undergoing chemotherapy exhibited relatively limited knowledge (mean score 12.07 ± 4.69, 60.4% of maximum) but maintained generally positive attitudes (mean score 35.89 ± 4.98, 79.8% of maximum) and moderately proactive practices (mean score 23.28 ± 4.12, 77.6% of maximum) toward nutritional management. This pattern of high motivation despite knowledge gaps suggests that educational interventions may find receptive audiences among lung cancer patients, though the content must address specific knowledge deficits identified in our study. When compared to previous studies, such as the investigation on liver cancer patients receiving immunotherapy, where patients demonstrated moderate knowledge and high attitude and practice scores (66.0, 82.5, and 76.8%, respectively) (15), our study displayed a comparable pattern of high attitude and practice levels despite suboptimal knowledge. This may reflect a shared recognition among cancer patients of the importance of nutritional care, even in the context of knowledge deficits, particularly in settings where clinical teams provide ongoing treatment support. In contrast, a large-scale cross-sectional study among patients with gastrointestinal cancer found that while over 80% had adequate nutritional knowledge, only 26.4 and 32.5% exhibited positive attitudes and proactive practices, respectively (14). This discrepancy may be attributed to differences in disease trajectory, treatment modality, or patient education. GI cancer patients often receive perioperative nutritional counseling, potentially boosting knowledge, yet may lack sustained reinforcement during long-term care, contributing to passive behavior. Conversely, chemotherapy-associated nutritional side effects in lung cancer may heighten patients’ motivation to actively engage in nutritional self-management, despite a lack of comprehensive understanding.

The associations identified among the KAP dimensions support a conceptual model in which knowledge and attitudes function jointly rather than in isolation. Correlational analyses revealed moderate links across domains, and structural modeling suggested that while knowledge directly influences practice, a substantial portion of its impact is mediated through attitude. Our structural equation modeling demonstrated that knowledge exerted both direct effects on practice (*β* = 0.345, *p* = 0.006) and indirect effects through attitude (*β* = 0.335, *p* = 0.014), with attitude also directly influencing practice (*β* = 0.558, *p* = 0.010). The total effect of knowledge on practice (*β* = 0.680, *p* = 0.007) suggests that approximately 49.3% of knowledge’s influence operates through attitudinal mediation. This finding has important clinical implications: purely didactic nutritional education may have limited impact if it does not also address patients’ motivational factors and perceived barriers. Interventions should therefore combine information provision with strategies to strengthen positive attitudes, address concerns about treatment burden, and build self-efficacy for nutritional self-management. The strong direct effect of attitude on practice (*β* = 0.558) further emphasizes that even when knowledge is adequate, behavioral change depends heavily on patients’ beliefs about the importance, feasibility, and benefits of nutritional management during chemotherapy. This mediation pattern is further supported by findings from liver cancer patients, where attitude scores were significantly correlated with both practice scores (*r* = 0.460, *p* < 0.001) and lifestyle scores (*r* = 0.486, *p* < 0.001), while knowledge was only moderately correlated with attitude (*r* = 0.105, *p* = 0.035) (15). The stronger influence of knowledge on attitude than on behavior may indicate that patients require a sustained cognitive foundation before behavioral adherence becomes consistent (25). At the same time, the model’s emphasis on indirect effects points to the inadequacy of purely informational interventions, particularly in populations managing complex clinical regimens.

Variation in KAP scores across demographic groups offers further insight into how health behaviors emerge in context. Urban residents, individuals who were married, and those exposed to formal nutritional education scored higher on nearly every measure. These sociodemographic patterns are consistently observed in other cancers, as demonstrated in liver cancer patients where urban residence was associated with higher attitude scores, living with someone was associated with higher attitude scores, and female gender was associated with higher lifestyle scores (15). These patterns are aligned with observations from studies in settings with differential access to healthcare resources, where geographic proximity to medical services and family-based support structures facilitate more active engagement with care protocols (26). It is also possible that the observed rural–urban differences reflect broader socioeconomic disparities, including access to nutritional resources, health services, and digital health information. Such structural inequalities may have influenced participants’ opportunities to acquire nutritional knowledge or engage in recommended practices, thereby introducing potential bias into the comparison between groups (27). Notably, participants who had received nutrition education from hospitals or other healthcare institutions demonstrated significantly higher KAP scores. This finding suggests that structured education, typically delivered by clinical dietitians or oncology nurses, plays a key role in improving patients’ nutritional literacy and self-management capacity.

Younger patients, those with lower education levels, and individuals at nutritional risk showed evident gaps in specific knowledge and behavioral aspects, reflecting disparities related to age, education, and nutritional status. Notably, patients without bachelor’s degrees scored significantly lower on technical knowledge items and showed less consistent weight monitoring behaviors. Similarly, patients at nutritional risk (NRS-2002 > 3) demonstrated lower knowledge of protein supplementation, less positive attitudes toward nutritional importance (A3), and poorer adherence to diet adjustment (P1) and balanced nutrition (P3, P4). These findings suggest that nutritional interventions should be stratified by education level and current nutritional status, with simplified, practical guidance for those with lower health literacy and intensified monitoring for those already at nutritional risk. Similar trends have been observed among cancer patients, as younger individuals often show lower adherence to sustained dietary management during treatment, while those with lower educational attainment exhibit poorer comprehension and application of nutritional guidance (28). Patients at nutritional risk were also less aware of protein requirements and less consistent in dietary monitoring, suggesting that malnutrition may further impede learning and adherence. These findings suggest that nutritional interventions should be adapted to patients’ age, education level, and health status to improve accessibility and effectiveness. Although knowledge and attitudes did not differ significantly between NRS-2002 groups, this pattern may be explained by the fact that both constructs primarily reflect cognitive and perceptual understanding, which is less immediately affected by current nutritional status. In contrast, practices require sustained behavioral engagement and physical capacity. Individuals with nutritional risk may experience fatigue, appetite loss, or treatment burden that directly hinder their ability to maintain recommended dietary behaviors or regular monitoring. As a result, differences in practice scores are more likely to emerge even when knowledge and attitudes remain comparable.

Although education level did not influence knowledge or practice scores to a statistically discernible extent, it was associated with attitudinal measures, especially among respondents holding a university degree. This finding departs slightly from expectations rooted in health literacy models, which often assume a more direct link between educational attainment and both informational and behavioral outcomes (29). One possible explanation is that attitudinal receptiveness to nutritional advice develops independently of procedural knowledge, reflecting broader orientations toward health that are shaped by educational culture rather than specific content exposure. A similar pattern was observed with clinical experience: patients with longer durations since diagnosis or more extensive treatment histories tended to report better knowledge and more consistent practices (30). These associations may reflect cumulative exposure to healthcare guidance, or they may represent a selection effect in which those who have remained in care longer have internalized key messages over time.

Specifically, our data showed that only 24.25% of participants were very familiar with recommended energy intake targets (25–30 kcal/kg/day), and only 30.1% clearly understood indications for parenteral nutrition. These findings align with previous research showing that cancer patients often possess general nutritional awareness but lack specific quantitative knowledge (15). The knowledge gap in technical nutritional parameters may explain why 14.1% of our participants had NRS-2002 scores indicating nutritional risk despite 79.8% expressing positive attitudes toward nutrition. Similar knowledge gaps have been identified in liver cancer patients, where the lowest knowledge scores were observed for questions about adverse reactions during immunotherapy (50.75%) and prompt identification of malnutrition symptoms (72.39%) (15). The uneven distribution of responses suggests that while some conceptual frameworks are absorbed, clinical specificity is not. Educational efforts may be constrained by time limitations during consultations or by the absence of accessible, tailored materials. It is also possible that clinicians themselves vary in how comprehensively they address nutritional topics, particularly in facilities without embedded dietary support (31).

Attitudinal responses were more consistently favorable, though not without areas of ambivalence. While 66.39% of participants reported being worried or very worried about malnutrition (A1), and 78.6% were concerned that malnutrition might affect chemotherapy outcomes (A3), only 74.58% expressed willingness to invest time and money for better nutritional management (A9), and 22.07% remained neutral about parenteral nutrition (A7). This disparity between concern and willingness to act suggests that perceived barriers—whether financial, procedural, or related to treatment complexity—may attenuate the translation of nutritional concerns into behavioral commitment. Our barrier analysis supports this interpretation: 72.6% cited limited knowledge, 64.7% reported complexity and time constraints, and 29.3% noted financial burden as obstacles to nutritional management. This pattern mirrors findings in liver cancer patients, where the lowest attitude score related to nutritional support significance (64.52%), while the highest score concerned maintaining positive mindset and quality sleep (93.28%). Additionally, practice scores revealed reluctance toward more invasive interventions, with only 39.53% expressing preparedness to receive enteral feeding if required. Responses related to parenteral nutrition and financial investment, for instance, were notably more reserved. This hesitancy may reflect prior clinical encounters, cultural conceptions of invasive treatment, or broader concerns about the financial burden of care (32). Even where beliefs about nutritional importance were strong, the perceived feasibility of specific actions appeared to remain uncertain for a sizable proportion of participants (33).

Practice data suggest moderate adherence to nutritional recommendations, with the most consistent behaviors reported in areas such as diet modification (69.73% always/often) and meal balance (73.58% always/often). However, other beneficial practices showed lower adherence: only 61.37% always/often used nutritional supplements (P5), and 12.21% rarely or never maintained regular physical activity (P6). Multivariate analysis revealed that practice scores were significantly associated with knowledge and attitude, suggesting that improving knowledge and reinforcing positive attitudes could enhance adherence to these more challenging behaviors. The lower adherence to supplement use and physical activity may reflect not only knowledge gaps but also treatment-related fatigue, financial constraints, and the cumulative burden of managing multiple self-care behaviors during chemotherapy (34). The importance of comprehensive practice implementation is emphasized by evidence from systematic reviews showing that nurse-led dietary interventions can achieve statistically significant improvements in fruit and vegetable intake (*p* = 0.009 and *p* = 0.003 in separate studies) and energy intake (*p* < 0.001) among cancer patients, demonstrating the potential for structured intervention programs (34). Reported barriers help clarify these trends: respondents cited not only informational gaps, but also time constraints and resource limitations. These findings are not unexpected in a patient population managing the cumulative effects of systemic treatment. Previous research in oncology settings has indicated that while patients often attempt to follow guidelines, their capacity to do so is influenced by energy levels, competing clinical demands, and the accessibility of supportive services (35).

The identification of physicians and digital platforms as key information sources reflects an evolving educational landscape. While the clinical setting remains central, an increasing proportion of patients report supplementing formal instruction with materials accessed through social media and online platforms. This diversification of information channels raises questions about content quality and standardization (36). It also suggests opportunities for outreach that are not currently structured within the clinical system, particularly in rural or under-resourced areas (37).

Several points of action follow from these observations. Nutritional services should be integrated more explicitly into the oncology care framework, with formalized roles for trained dietitians and routine screening for nutritional risk. Given the demonstrated effectiveness of nurse-led dietary interventions in cancer care, where nurses successfully delivered dietary counseling that improved fruit and vegetable intake and energy consumption, healthcare systems should consider expanding nurses’ roles beyond traditional nutritional screening to include active dietary counseling and intervention delivery (34). Patients should receive repeated exposure to key messages over the course of treatment, rather than at isolated points of care (31). In settings where in-person education is less feasible, digital platforms could be adapted to disseminate vetted, evidence-based information (38).

To address geographical disparities, mobile and telehealth-based nutrition services may help extend access to remote populations. The significant urban–rural disparities observed across multiple cancer types, including both lung and liver cancer populations, highlight the urgent need for these alternative delivery methods to ensure equitable access to nutritional support services. Structural supports for clinician training should be expanded, ensuring that oncologists and nurses receive baseline competencies in nutritional counseling (33). Health system administrators can support these efforts by incorporating nutrition metrics into quality monitoring systems, enabling iterative improvements and ensuring alignment between clinical priorities and institutional practice (38). These findings highlight several practical implications for clinical operations. First, given that 14.1% of participants had NRS-2002 scores indicating nutritional risk and that nutritional risk was independently associated with poorer practice scores, routine nutritional screening at each chemotherapy cycle is warranted. Second, because participants who received nutrition education from healthcare institutions scored significantly higher on knowledge, attitude, and practice, structured nutritional counseling should be systematically integrated into chemotherapy care pathways. Third, our finding that 72.6% of patients cited limited knowledge as the primary barrier to nutritional management indicates clear unmet educational needs that could be addressed through nurse-led or dietitian-led interventions during routine clinical visits (34). Integrating standardized educational materials into existing clinical workflows or digital platforms may further support consistent patient guidance. Such operational approaches would allow nutritional management to be implemented with minimal disruption while ensuring that patients at risk receive timely support.

Adequate nutritional status plays a crucial role in treatment tolerance, immune function, and overall recovery among patients with lung cancer. Evidence indicates that malnutrition and weight loss are associated with higher chemotherapy toxicity, increased postoperative complications, poorer quality of life, and reduced survival (39). Although this study did not collect direct clinical outcome data, the observed gaps in nutritional knowledge and practices highlight an important area for intervention that may ultimately influence these outcomes. Future longitudinal research integrating objective nutritional and clinical indicators is warranted to clarify these relationships.

This study has several limitations. First, its single-center cross-sectional design limits the ability to establish causal relationships between knowledge, attitudes, and practices. Second, as the study was conducted at a single center, the findings may not be generalizable to broader or more diverse lung cancer populations. Future studies should therefore be conducted across multiple regions and medical centers to improve external validity and ensure broader representativeness. Third, the use of convenience sampling through online recruitment may introduce selection bias, as participants with greater health awareness were more likely to participate. Additionally, recruitment through WeChat may have excluded individuals with limited digital access or lower socioeconomic status, further contributing to selection bias and reducing the representativeness of the sample. Future studies should consider a multi-channel recruitment strategy that incorporates hospital-based enrollment and community outreach to better capture underrepresented patient groups. Fourth, self-reported data may be subject to recall bias or social desirability bias, potentially affecting the accuracy of responses related to nutritional behaviors. In particular, the reliance on self-reported practices may have led to an overestimation of adherence to recommended dietary behaviors or physical activity, thereby influencing the observed strength of associations between knowledge, attitudes, and practices. These biases should be taken into account when interpreting the direction and magnitude of the relationships identified in the KAP model. Fifth, the knowledge items differed in difficulty, and some questions, such as those concerning recommended energy intake, were more challenging for respondents. Future versions should be refined using patient feedback and readability testing. Sixth, nutritional risk was assessed using NRS-2002 rather than direct nutritional status indicators, as anthropometric and biochemical assessments were not feasible in an online format. Comprehensive nutritional assessments, including anthropometric, biochemical, and dietary measures, are needed in future research to better contextualize KAP findings and strengthen clinical interpretation. Lastly, the absence of clinical outcome data limits the ability to determine how nutritional KAP influences treatment tolerance, complications, or survival. Based on these findings, future longitudinal studies should incorporate comprehensive nutritional and clinical assessments and evaluate whether KAP-informed educational interventions delivered collaboratively by dietitians, nurses, and oncologists can effectively improve patients’ nutritional status and clinical outcomes. Integrating digital platforms could further enhance these multidisciplinary efforts to support nutritional literacy, adherence to dietary guidance, and treatment success during chemotherapy.

## Conclusion

In conclusion, although patients with lung cancer undergoing chemotherapy exhibited generally positive attitudes and moderately proactive nutritional practices, their overall level of knowledge remained suboptimal, potentially limiting the effectiveness of their dietary behaviors. Maintaining adequate nutritional status is essential for optimizing treatment tolerance, minimizing complications, and improving overall prognosis in lung cancer patients. The validated questionnaire in this study provides a useful tool to identify educational gaps and inform structured, multidisciplinary interventions aimed at improving both nutritional literacy and clinical prognosis. Future prospective studies should evaluate whether interventions based on KAP findings lead to measurable improvements in nutritional status, treatment tolerance, and survival outcomes.

## Data Availability

The original contributions presented in the study are included in the article/Supplementary material, further inquiries can be directed to the corresponding author.

## Footnotes

1
https://www.wjx.cn/

## Supplementary material

SUPPLEMENTARY FIGURE S1 Confirmatory factor analysis (CFA) of the knowledge-attitude-practice (KAP) questionnaire.
