# Supplementary figures and images for: Knowledge, attitudes, and practices of lung cancer patients regarding nutritional management during chemotherapy
Source: Front Nutr. 2026 Jan 21;13:1678612. doi: 10.3389/fnut.2026.1678612 (PMC12867779; doi:10.3389/fnut.2026.1678612)

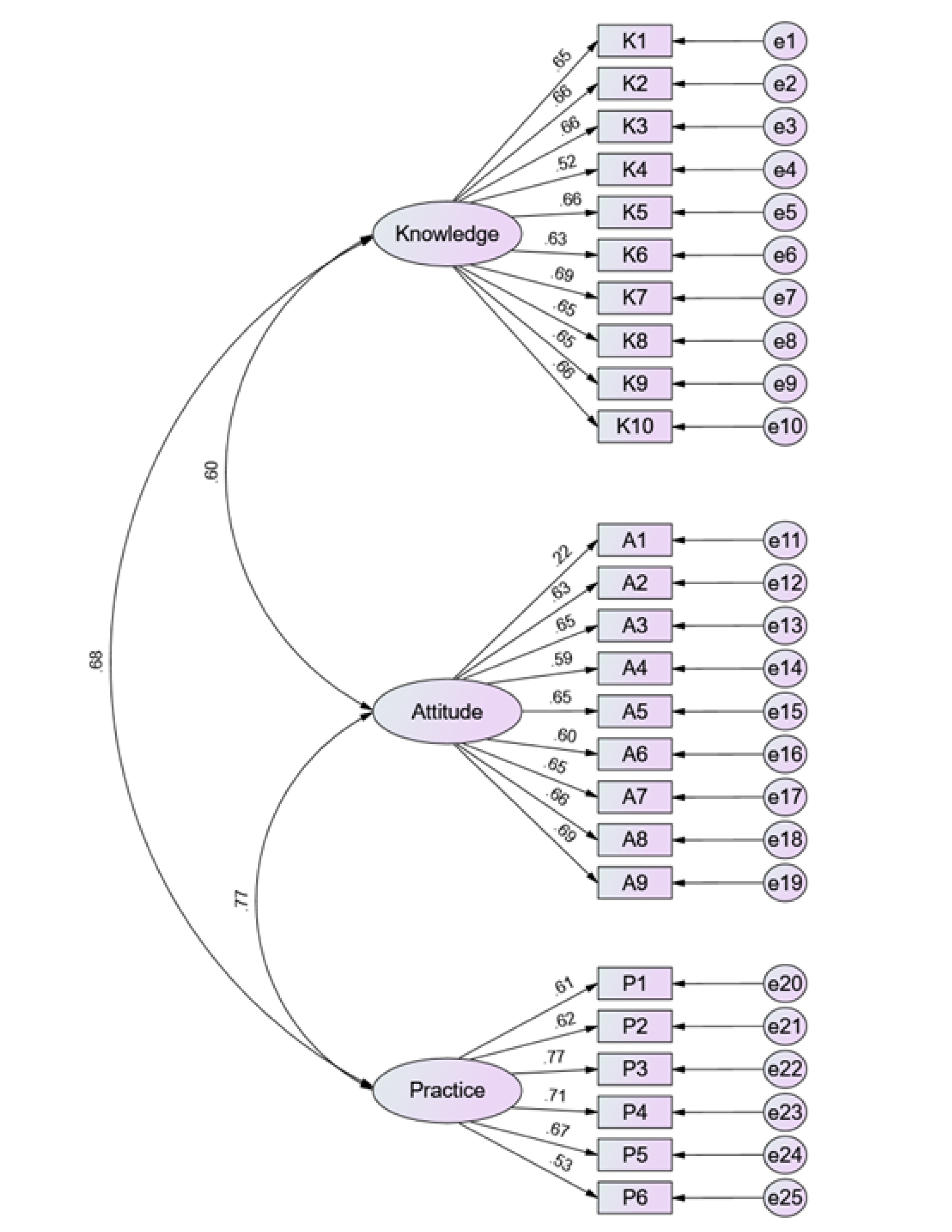

Supplement: Supplementary file 1 [file Image_1.tif]
