# Supplementary material for: Knowledge, attitudes, and practices of lung cancer patients regarding nutritional management during chemotherapy
Source: Front Nutr. 2026 Jan 21;13:1678612. doi: 10.3389/fnut.2026.1678612 (PMC12867779; doi:10.3389/fnut.2026.1678612)
Supplement: Supplementary file 2 [file Table_1.docx]

# **Table S1. Model fit indices for confirmatory factor analysis (CFA) of the Knowledge–Attitude–Practice (KAP) questionnaire.**

| Indicators | Reference | Actual |
| --- | --- | --- |
| CMIN/DF | 1-3: Excellent, 3-5: Good | 2.823 |
| RMSEA | <0.08: Good | 0.055 |
| IFI | >0.8: Good | 0.910 |
| TLI | >0.8: Good | 0.900 |
| CFI | >0.8: Good | 0.910 |

CMIN/DF, chi-square minimum/degree of freedom ratio; RMSEA, root mean square error of approximation; IFI, incremental fit index; TLI, Tucker–Lewis index; CFI, comparative fit index.

**Table S2. Standardized factor loadings and parameter estimates from the confirmatory factor analysis (CFA) of the Knowledge–Attitude–Practice (KAP) questionnaire.**

|  |  |  | **Estimate** | **Standardized Estimate** | **S.E.** | **C.R.** | **P** |
| --- | --- | --- | --- | --- | --- | --- | --- |
| K1 | <--- | Knowledge | 1.000 | 0.652 |  |  |  |
| K2 | <--- | Knowledge | 1.152 | 0.660 | 0.082 | 13.974 | <0.001 |
| K3 | <--- | Knowledge | 1.218 | 0.664 | 0.087 | 14.048 | <0.001 |
| K4 | <--- | Knowledge | 1.001 | 0.516 | 0.089 | 11.278 | <0.001 |
| K5 | <--- | Knowledge | 1.194 | 0.661 | 0.085 | 13.998 | <0.001 |
| K6 | <--- | Knowledge | 1.187 | 0.627 | 0.089 | 13.383 | <0.001 |
| K7 | <--- | Knowledge | 1.263 | 0.695 | 0.087 | 14.593 | <0.001 |
| K8 | <--- | Knowledge | 1.130 | 0.646 | 0.082 | 13.725 | <0.001 |
| K9 | <--- | Knowledge | 1.158 | 0.654 | 0.083 | 13.875 | <0.001 |
| K10 | <--- | Knowledge | 1.192 | 0.658 | 0.085 | 13.943 | <0.001 |
| A1 | <--- | Attitude | 1.000 | 0.225 |  |  |  |
| A2 | <--- | Attitude | 2.424 | 0.631 | 0.480 | 5.046 | <0.001 |
| A3 | <--- | Attitude | 2.424 | 0.646 | 0.479 | 5.060 | <0.001 |
| A4 | <--- | Attitude | 2.366 | 0.588 | 0.473 | 5.000 | <0.001 |
| A5 | <--- | Attitude | 2.432 | 0.652 | 0.480 | 5.065 | <0.001 |
| A6 | <--- | Attitude | 2.351 | 0.601 | 0.469 | 5.015 | <0.001 |
| A7 | <--- | Attitude | 2.549 | 0.651 | 0.503 | 5.065 | <0.001 |
| A8 | <--- | Attitude | 2.373 | 0.657 | 0.468 | 5.069 | <0.001 |
| A9 | <--- | Attitude | 2.493 | 0.687 | 0.489 | 5.094 | <0.001 |
| P1 | <--- | Practice | 1.000 | 0.612 |  |  |  |
| P2 | <--- | Practice | 1.080 | 0.621 | 0.087 | 12.394 | <0.001 |
| P3 | <--- | Practice | 1.250 | 0.773 | 0.086 | 14.465 | <0.001 |
| P4 | <--- | Practice | 1.146 | 0.713 | 0.084 | 13.710 | <0.001 |
| P5 | <--- | Practice | 1.216 | 0.669 | 0.093 | 13.096 | <0.001 |
| P6 | <--- | Practice | 0.918 | 0.531 | 0.084 | 10.948 | <0.001 |

S.E., Standard Error; C.R., Critical Ratio.

**Table S3. Correlation analysis**

|  | Knowledge | Attitude | Practice |
| --- | --- | --- | --- |
| Knowledge | 1 |  |  |
| Attitude | 0.520 (P<0.001) | 1 |  |
| Practice | 0.570 (P<0.001) | 0.546 (P<0.001) | 1 |

**Table S4. SEM fit indicators**

| **Model Fit Indicators** | **Ref.** | **Measured results** |
| --- | --- | --- |
| **CMIN/DF** | 1-3 excellent，3-5 good | 2.823 |
| **RMSEA** | <0.08 good | 0.055 |
| **IFI** | >0.8 good | 0.910 |
| **TLI** | >0.8 good | 0.900 |
| **CFI** | >0.8 good | 0.910 |

CMIN/DF, chi-square minimum/degree of freedom ratio; RMSEA, root mean square error of approximation; IFI, incremental fit index; TLI, Tucker–Lewis index; CFI, comparative fit index.

**Table S5. Univariate and multivariate linear regression analyses of demographic and clinical factors associated with knowledge scores among patients with lung cancer undergoing chemotherapy.**

| Knowledge | Univariate linear regression | |  | Multivariate linear regression | |
| --- | --- | --- | --- | --- | --- |
|  | β (95%CI) | P |  | β (95%CI) | P |
| **Age (years)** |  |  |  |  |  |
| 18-39 | Ref |  |  |  |  |
| 40-60 | 0.304 (-0.647,1.256) | 0.530 |  |  |  |
| 61 and more | 0.257 (-0.712,1.225) | 0.603 |  |  |  |
| **Gender** |  |  |  |  |  |
| Male | Ref |  |  |  |  |
| Female | 0.092 (-0.670,0.855) | 0.812 |  |  |  |
| **Residence** |  |  |  |  |  |
| Rural | Ref |  |  | Ref |  |
| Urban | 1.210 (0.439,1.980) | 0.002 |  | 0.713 (-0.070,1.495) | 0.074 |
| **Education** |  |  |  |  |  |
| Junior high school and below | Ref |  |  | Ref |  |
| High school / Technical secondary school | 0.677 (-0.331,1.685) | 0.188 |  | 0.323 (-0.661,1.307) | 0.519 |
| Associate degree | 0.335 (-0.739,1.409) | 0.541 |  | 0.387 (-0.691,1.465) | 0.481 |
| Bachelor's degree and above | 1.018 (0.007,2.028) | 0.048 |  | 1.612 (0.495,2.729) | 0.005 |
| **Marital status** |  |  |  |  |  |
| Unmarried | Ref |  |  | Ref |  |
| Married | 1.737 (0.825,2.648) | <0.001 |  | 1.808 (0.793,2.823) | <0.001 |
| **Monthly income per capita (CNY)** |  |  |  |  |  |
| <2000 | Ref |  |  |  |  |
| 2000-5000 | 0.837 (-0.305,1.980) | 0.151 |  |  |  |
| 5000-10000 | 0.460 (-0.751,1.671) | 0.456 |  |  |  |
| >10000 | 1.663 (-0.049,3.376) | 0.057 |  |  |  |
| **Time since lung cancer diagnosis** |  |  |  |  |  |
| Within 1 month | Ref |  |  | Ref |  |
| 1-3 months | 0.677 (-0.864,2.219) | 0.388 |  | 0.417 (-1.047,1.882) | 0.576 |
| 3-6 months | 0.847 (-0.506,2.200) | 0.220 |  | -0.166 (-1.482,1.150) | 0.804 |
| 6 months -1 year | 0.724 (-0.621,2.070) | 0.291 |  | -0.027 (-1.319,1.265) | 0.967 |
| 1-3 years | 1.460 (0.124,2.795) | 0.032 |  | 1.057 (-0.269,2.382) | 0.118 |
| More than 3 years | 2.550 (1.061,4.040) | <0.001 |  | 1.838 (0.370,3.305) | 0.014 |
| **History of surgery** |  |  |  |  |  |
| Yes | 0.508 (-0.246,1.261) | 0.186 |  |  |  |
| No | Ref |  |  |  |  |
| **History of radiotherapy** |  |  |  |  |  |
| Yes | -0.313 (-1.067,0.442) | 0.416 |  |  |  |
| No | Ref |  |  |  |  |
| **History of chemotherapy** |  |  |  |  |  |
| Yes | 1.425 (0.671,2.179) | <0.001 |  | 1.318 (0.575,2.060) | <0.001 |
| No | Ref |  |  | Ref |  |
| **BMI** |  |  |  |  |  |
| <18.5 | 0.203 (-0.839,1.245) | 0.702 |  |  |  |
| 18.5-24.9 | Ref |  |  |  |  |
| >24.9 | -0.762 (-1.674,0.149) | 0.101 |  |  |  |
| **Family History of Lung Cancer (First-Degree Relatives)** |  |  |  |  |  |
| Yes | Ref |  |  |  |  |
| No | -0.355 (-1.177,0.467) | 0.397 |  |  |  |
| **Education from a hospital or other healthcare institutions** |  |  |  |  |  |
| Yes | Ref |  |  | Ref |  |
| No | -2.401 (-3.150,-1.651) | <0.001 |  | -2.306 (-3.062,-1.550) | <0.001 |
| **NRS-2002** |  |  |  |  |  |
| ≤3 | Ref |  |  |  |  |
| >3 | -0.509 (-1.594,0.576) | 0.357 |  |  |  |

**Table S6. Univariate and multivariate linear regression analyses of demographic and clinical factors associated with attitude scores among patients with lung cancer undergoing chemotherapy.**

| Attitude | Univariate linear regression | |  | Multivariate linear regression | |
| --- | --- | --- | --- | --- | --- |
|  | β (95%CI) | P |  | β (95%CI) | P |
| **Knowledge** | 0.534 (0.460,0.608) | <0.001 |  | 0.47 (0.394,0.545) | <0.001 |
| **Age (years)** |  |  |  |  |  |
| 18-39 | Ref |  |  |  |  |
| 40-60 | 0.817 (-0.191,1.826) | 0.112 |  |  |  |
| 61 and more | 0.734 (-0.292,1.760) | 0.161 |  |  |  |
| **Gender** |  |  |  |  |  |
| Male | Ref |  |  |  |  |
| Female | -0.181 (-0.990,0.629) | 0.661 |  |  |  |
| **Residence** |  |  |  |  |  |
| Rural | Ref |  |  |  |  |
| Urban | 0.726 (-0.096,1.549) | 0.083 |  |  |  |
| **Education** |  |  |  |  |  |
| Junior high school and below | Ref |  |  | Ref |  |
| High school / Technical secondary school | -0.262 (-1.324,0.800) | 0.628 |  | -0.742 (-1.651,0.168) | 0.11 |
| Associate degree | -1.182 (-2.314,-0.050) | 0.041 |  | -1.151 (-2.136,-0.165) | 0.022 |
| Bachelor's degree and above | 1.104 (0.039,2.169) | 0.042 |  | 0.627 (-0.393,1.646) | 0.228 |
| **Marital status** |  |  |  |  |  |
| Unmarried | Ref |  |  | Ref |  |
| Married | 1.247 (0.273,2.221) | 0.012 |  | 0.934 (-0.002,1.869) | 0.050 |
| **Monthly income per capita (CNY)** |  |  |  |  |  |
| <2000 | Ref |  |  |  |  |
| 2000-5000 | 0.665 (-0.550,1.881) | 0.283 |  |  |  |
| 5000-10000 | 0.255 (-1.033,1.543) | 0.698 |  |  |  |
| >10000 | 1.186 (-0.636,3.008) | 0.201 |  |  |  |
| **Time since lung cancer diagnosis** |  |  |  |  |  |
| Within 1 month | Ref |  |  |  |  |
| 1-3 months | 0.327 (-1.321,1.975) | 0.697 |  |  |  |
| 3-6 months | -0.275 (-1.722,1.172) | 0.709 |  |  |  |
| 6 months -1 year | -0.112 (-1.550,1.326) | 0.878 |  |  |  |
| 1-3 years | 0.585 (-0.843,2.013) | 0.421 |  |  |  |
| More than 3 years | 1.215 (-0.377,2.808) | 0.134 |  |  |  |
| **History of surgery** |  |  |  |  |  |
| Yes | 1.102 (0.306,1.899) | 0.007 |  | 0.649 (-0.042,1.341) | 0.066 |
| No | Ref |  |  | Ref |  |
| **History of radiotherapy** |  |  |  |  |  |
| Yes | -0.984 (-1.781, -0.186) | 0.016 |  | -0.717 (-1.401, -0.033) | 0.040 |
| No | Ref |  |  | Ref |  |
| **History of chemotherapy** |  |  |  |  |  |
| Yes | 0.639 (-0.169,1.447) | 0.121 |  |  |  |
| No | Ref |  |  |  |  |
| **BMI** |  |  |  |  |  |
| <18.5 | 1.101 (0.001,2.203) | 0.049 |  | 0.914 (-0.024,1.852) | 0.056 |
| 18.5-24.9 | Ref |  |  | Ref |  |
| >24.9 | -0.815 (-1.778,0.149) | 0.097 |  | -0.433 (-1.257,0.39) | 0.302 |
| **Family History of Lung Cancer (First-Degree Relatives)** |  |  |  |  |  |
| Yes | Ref |  |  |  |  |
| No | 0.380 (-0.492,1.253) | 0.392 |  |  |  |
| **Education from a hospital or other healthcare institutions** |  |  |  |  |  |
| Yes | Ref |  |  | Ref |  |
| No | -2.543 (-3.338,-1.746) | <0.001 |  | -1.297 (-2.029,-0.565) | 0.001 |
| **NRS-2002** |  |  |  |  |  |
| ≤3 | Ref |  |  | Ref |  |
| >3 | -1.726 (-2.870, -0.582) | 0.003 |  | -1.3 (-2.294, -0.306) | 0.010 |

**Table S7. Univariate and multivariate linear regression analyses of demographic and clinical factors associated with practice score among patients with lung cancer undergoing chemotherapy.**

| Practice | Univariate linear regression | |  | Multivariate linear regression | |
| --- | --- | --- | --- | --- | --- |
|  | β (95%CI) | P |  | β (95%CI) | P |
| **Knowledge** | 0.498 (0.440,0.556) | <0.001 |  | 0.285 (0.225,0.345) | <0.001 |
| **Attitude** | 0.503 (0.450,0.556) | <0.001 |  | 0.344 (0.287,0.401) | <0.001 |
| **Age (years)** |  |  |  |  |  |
| 18-39 | Ref |  |  | Ref |  |
| 40-60 | 0.854 (0.023,1.684) | 0.044 |  | -0.197 (-0.927,0.534) | 0.597 |
| 61 and more | 1.035 (0.190,1.880) | 0.016 |  | -0.327 (-1.09,0.436) | 0.401 |
| **Gender** |  |  |  |  |  |
| Male | Ref |  |  |  |  |
| Female | 0.116 (-0.553,0.784) | 0.734 |  |  |  |
| **Residence** |  |  |  |  |  |
| Rural | Ref |  |  | Ref |  |
| Urban | 1.066 (0.391,1.742) | 0.002 |  | 0.520 (0.008,1.032) | 0.047 |
| **Education** |  |  |  |  |  |
| Junior high school and below | Ref |  |  |  |  |
| High school / Technical secondary school | 0.430 (-0.455,1.316) | 0.340 |  |  |  |
| Associate degree | -0.100 (-1.043,0.844) | 0.836 |  |  |  |
| Bachelor's degree and above | 0.472 (-0.416,1.359) | 0.297 |  |  |  |
| **Marital status** |  |  |  |  |  |
| Unmarried | Ref |  |  | Ref |  |
| Married | 1.738 (0.942,2.535) | <0.001 |  | 0.540 (-0.170,1.250) | 0.136 |
| **Monthly income per capita (CNY)** |  |  |  |  |  |
| <2000 | Ref |  |  |  |  |
| 2000-5000 | 0.442 (-0.559,1.444) | 0.386 |  |  |  |
| 5000-10000 | -0.346 (-1.407,0.715) | 0.522 |  |  |  |
| >10000 | 0.813 (-0.688,2.314) | 0.288 |  |  |  |
| **Time since lung cancer diagnosis** |  |  |  |  |  |
| Within 1 month | Ref |  |  | Ref |  |
| 1-3 months | 0.784 (-0.546,2.115) | 0.247 |  | 0.53 (-0.452,1.511) | 0.289 |
| 3-6 months | 0.391 (-0.778,1.559) | 0.512 |  | 0.266 (-0.627,1.159) | 0.559 |
| 6 months -1 year | 0.884 (-0.277,2.045) | 0.135 |  | 0.802 (-0.086,1.689) | 0.076 |
| 1-3 years | 2.351 (1.198,3.504) | <0.001 |  | 1.651 (0.74,2.563) | <0.001 |
| More than 3 years | 2.637 (1.351,3.922) | <0.001 |  | 1.522 (0.486,2.557) | 0.004 |
| **History of surgery** |  |  |  |  |  |
| Yes | 0.185 (-0.476,0.847) | 0.582 |  |  |  |
| No | Ref |  |  |  |  |
| **History of radiotherapy** |  |  |  |  |  |
| Yes | -1.046 (-1.7703,-0.389) | 0.002 |  | -0.625 (-1.109,-0.142) | 0.011 |
| No | Ref |  |  | Ref |  |
| **History of chemotherapy** |  |  |  |  |  |
| Yes | 0.506 (-0.161,1.174) | 0.137 |  |  |  |
| No | Ref |  |  |  |  |
| **BMI** |  |  |  |  |  |
| <18.5 | 0.970 (0.059,1.881) | 0.037 |  | 0.641 (-0.028,1.310) | 0.060 |
| 18.5-24.9 | Ref |  |  | Ref |  |
| >24.9 | -0.416 (-1.213,0.382) | 0.306 |  | 0.162 (-0.424,0.749) | 0.587 |
| **Family History of Lung Cancer (First-Degree Relatives)** |  |  |  |  |  |
| Yes | Ref |  |  |  |  |
| No | 0.619 (-0.100,1.338) | 0.092 |  |  |  |
| **Education from a hospital or other healthcare institutions** |  |  |  |  |  |
| Yes | Ref |  |  | Ref |  |
| No | -1.265 (-1.936,-0.594) | <0.001 |  | 0.139 (-0.392,0.669) | 0.608 |
| **NRS-2002** |  |  |  |  |  |
| ≤3 | Ref |  |  | Ref |  |
| >3 | -1.783 (-2.724,-0.842) | <0.001 |  | -0.730 (-1.469,0.010) | 0.053 |
